# 3-*O*-Benzhydryl-2,5-dide­oxy-2,5-imino-2-*C*-methyl-l-lyxono-1,4-lactone

**DOI:** 10.1107/S1600536808027888

**Published:** 2008-09-06

**Authors:** Filipa P. da Cruz, K. Victoria Booth, George W. J. Fleet, David J. Watkin

**Affiliations:** a Department of Organic Chemistry, Chemical Research Laboratory, University of Oxford, Mansfield Road, Oxford OX1 3TA, England; b Department of Chemical Crystallography, Chemical Research Laboratory, University of Oxford, Mansfield Road, Oxford OX1 3TA, England

## Abstract

The title bicyclic lactone, C_19_H_19_NO_3_, is an inter­mediate in the synthesis of chiral α-methyl­prolines and branched *C*-methyl pyrrolidines; the absolute configuration was determined by the use of d-erythronolactone as the starting material. It exhibits no unusual crystal packing features, and each mol­ecule acts as a donor and acceptor for one C—H⋯O hydrogen bond.

## Related literature

For use of carbohydrates in synthesis see: Monneret & Florent (1994 ▶); Ireland *et al.* (1983 ▶); Hotchkiss *et al.* (2006 ▶, 2007*a* ▶,*b* ▶); Dukhan *et al.* (2005 ▶); Rao *et al.* (2008 ▶); Punzo *et al.* (2005*a* ▶,*b* ▶); Da Cruz *et al.* (2008 ▶). For related crystallographic literature see: Larson (1970 ▶); Prince (1982 ▶); Watkin (1994 ▶). 
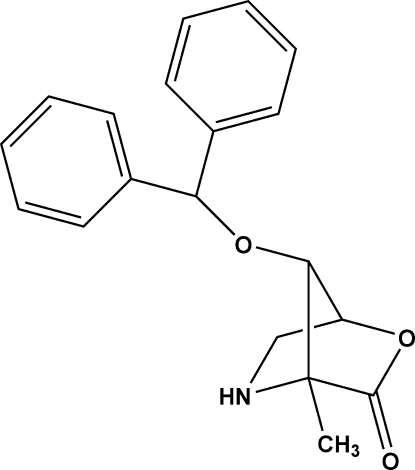


## Experimental

### 

#### Crystal data

C_19_H_19_NO_3_
*M* *_r_* = 309.36
Orthorhombic, 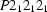

*a* = 9.0336 (2) Å
*b* = 10.0498 (2) Å
*c* = 17.5941 (4) Å
*V* = 1597.30 (6) Å^3^
*Z* = 4
Mo *K*α radiation
μ = 0.09 mm^−1^
*T* = 150 K
0.30 × 0.25 × 0.25 mm

#### Data collection

Nonius KappaCCD area-detector diffractometer
Absorption correction: multi-scan (*DENZO*/*SCALEPACK*; Otwinowski & Minor, 1997 ▶) *T*_min_ = 0.94, *T*_max_ = 0.98
25603 measured reflections
2071 independent reflections
1411 reflections with *I* > 2σ(*I*)
*R*_int_ = 0.053

#### Refinement

*R*[*F* ^2^ > 2σ(*F* ^2^)] = 0.029
*wR*(*F* ^2^) = 0.101
*S* = 0.86
2071 reflections
212 parameters
H atoms treated by a mixture of independent and constrained refinement
Δρ_max_ = 0.21 e Å^−3^
Δρ_min_ = −0.21 e Å^−3^

### 

Data collection: *COLLECT* (Nonius, 1997-2001 ▶).; cell refinement: *DENZO*/*SCALEPACK* (Otwinowski & Minor, 1997 ▶); data reduction: *DENZO*/*SCALEPACK*; program(s) used to solve structure: *SIR92* (Altomare *et al.*, 1994 ▶); program(s) used to refine structure: *CRYSTALS* (Betteridge *et al.*, 2003 ▶); molecular graphics: *CAMERON* (Watkin *et al.*, 1996 ▶); software used to prepare material for publication: *CRYSTALS*.

## Supplementary Material

Crystal structure: contains datablocks I. DOI: 10.1107/S1600536808027888/cs2089sup1.cif

Structure factors: contains datablocks I. DOI: 10.1107/S1600536808027888/cs2089Isup2.hkl

Additional supplementary materials:  crystallographic information; 3D view; checkCIF report

## Figures and Tables

**Table 1:** Hydrogen-bond geometry (Å, °)

*D*—H⋯*A*	*D*—H	H⋯*A*	*D*⋯*A*	*D*—H⋯*A*
C20—H201⋯O10^i^	0.93	2.36	3.293 (3)	174

Symmetry code: (i) 
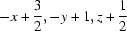
.

**Fig. 1.**
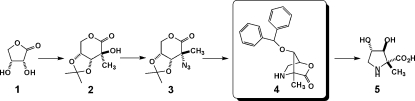
Synthetic scheme.

**Fig. 2.**
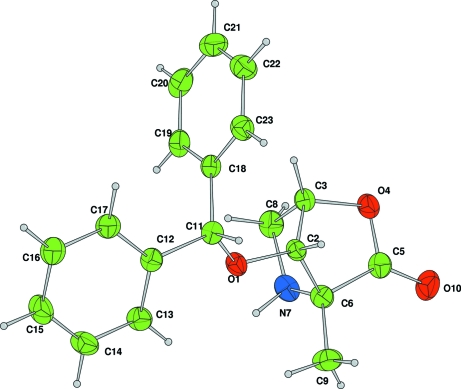
The title compound with displacement ellipsoids drawn at the 50% probability level. H atoms are shown as spheres of arbitary radius.

**Fig. 3.**
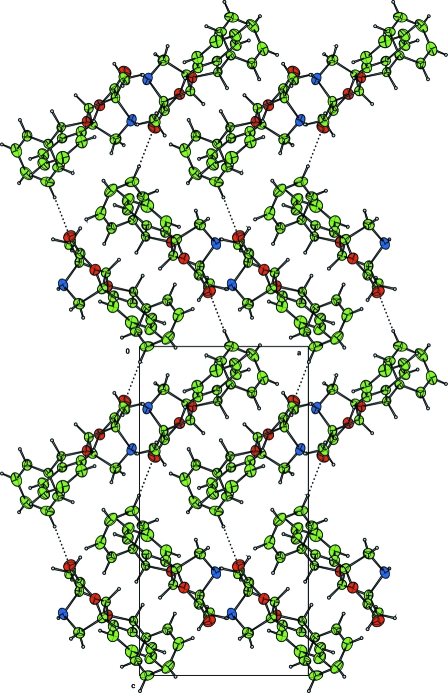
Packing diagram showing hydrogen bonded chains running parallel to the a-axis.

## supplementary crystallographic information

### Comment

Carbon-branched sugar lactones have hitherto been rarely used for the synthesis of enantiopure chiral targets (Monneret & Florent, 1994; Ireland *et al.*, 1983). 2-*C*-Methyl-D-ribonolactone has become readily available in large amounts (Hotchkiss *et al.*, 2007*a*) and has been used in the synthesis of branched α-*C*-nucleosides (Dukhan *et al.*, 2005), 4-*C*-methylpentuloses (Rao *et al.*, 2008) and branched imino sugars (Hotchkiss *et al.*, 2007*b*). Derivatives of 2-*C*-methyl-D-arabinonolactone, such as 2, are accessible from D-erythronolactone 1 by addition of methyl magnesium bromide followed by further reaction with sodium cyanide (Hotchkiss *et al.*, 2006; Punzo *et al.*, 2005*a*). The tertiary alcohol 2 may be efficiently converted into the *ribo*-azide 3, the structure of which has been confirmed by X-ray crystallographic analysis (Da Cruz *et al.*, 2008; Punzo *et al.*, 2005*b*). The relative stereochemistry of 4 is firmly established in this paper by X-ray crystallographic analysis and the absolute configuration is defined by the use of D-erythronolactone 1 as the starting material.

The title compound exhibits no unusual crystal packing features. Each molecule acts as a donor and acceptor for one hydrogen bond, forming chains approximately parallel to the *a*-axis. A suggested hydrogen bond [N7 - H1 - O10] has been ignored in the packing diagram as it exceeds the limits of standard hydrogen bond length (2.52 Å)

### Experimental

The title compound was recrystallized from cyclohexane and diethyl ether: m.p. 116–118°C; [α]_D_^21^ -26.0 (*c*, 1.0 in MeCN).

### Refinement

In the absence of significant anomalous scattering, Friedel pairs were merged. The H atoms were all located in a difference map, but those attached to carbon atoms were repositioned geometrically. The H atoms were initially refined with soft restraints on the bond lengths and angles to regularize their geometry (C—H in the range 0.93–0.98, N—H in the range 0.86–0.89 N—H to 0.86 O—H = 0.82 Å) and *U*_iso_(H) (in the range 1.2–1.5 times *U*_eq_ of the parent atom), after which the positions were refined with riding constraints.

### Crystal data

**Table:**

C_19_H_19_NO_3_	*F*(000) = 656
*M**_r_* = 309.36	*D*_x_ = 1.286 Mg m^−^^3^
Orthorhombic, *P*2_1_2_1_2_1_	Mo *K*α radiation, λ = 0.71073 Å
Hall symbol: P 2ac 2ab	Cell parameters from 6711 reflections
*a* = 9.0336 (2) Å	θ = 5–27°
*b* = 10.0498 (2) Å	µ = 0.09 mm^−^^1^
*c* = 17.5941 (4) Å	*T* = 150 K
*V* = 1597.30 (6) Å^3^	Block, colourless
*Z* = 4	0.30 × 0.25 × 0.25 mm

### Data collection

**Table:**

Nonius KappaCCD area-detector diffractometer	1411 reflections with *I* > 2σ(*I*)
graphite	*R*_int_ = 0.053
ω scans	θ_max_ = 27.5°, θ_min_ = 5.2°
Absorption correction: multi-scan (DENZO/SCALEPACK; Otwinowski & Minor, 1997)	*h* = −11→11
*T*_min_ = 0.94, *T*_max_ = 0.98	*k* = −13→12
25603 measured reflections	*l* = −22→22
2071 independent reflections	

### Refinement

**Table:**

Refinement on *F*^2^	Hydrogen site location: inferred from neighbouring sites
Least-squares matrix: full	H atoms treated by a mixture of independent and constrained refinement
*R*[*F*^2^ > 2σ(*F*^2^)] = 0.029	Method, part 1, Chebychev polynomial, (Watkin, 1994) [*w*eight] = 1.0/[A_0_*T_0_(x) + A_1_*T_1_(x) ··· + A_n-1_]*T_n-1_(x)] where A_i_ are the Chebychev coefficients listed belo*w* and x = *F* /*F*max Method = Robust Weighting (*P*rince, 1982) W = [*w*eight] * [1-(delta*F*/6*sigma*F*)^2^]^2^ A_i_ are: 16.5 25.4 13.4 3.97
*wR*(*F*^2^) = 0.101	(Δ/σ)_max_ = 0.000186
*S* = 0.86	Δρ_max_ = 0.21 e Å^−^^3^
2071 reflections	Δρ_min_ = −0.21 e Å^−^^3^
212 parameters	Extinction correction: Larson (1970), Equation 22
0 restraints	Extinction coefficient: 420 (70)
Primary atom site location: structure-invariant direct methods	

### Fractional atomic coordinates and isotropic or equivalent isotropic displacement parameters (Å^2^)

**Table:**

	*x*	*y*	*z*	*U*_iso_*/*U*_eq_	
O1	0.68597 (16)	0.81026 (14)	0.31734 (9)	0.0272	
C2	0.6994 (2)	0.69016 (19)	0.27608 (12)	0.0242	
C3	0.7190 (3)	0.5647 (2)	0.32511 (13)	0.0286	
O4	0.76321 (17)	0.46691 (14)	0.26720 (9)	0.0310	
C5	0.8514 (3)	0.5332 (2)	0.21745 (12)	0.0301	
C6	0.8569 (2)	0.6776 (2)	0.24385 (12)	0.0281	
N7	0.9525 (2)	0.6653 (2)	0.31282 (12)	0.0325	
C8	0.8577 (3)	0.5979 (2)	0.37011 (13)	0.0337	
C9	0.9106 (3)	0.7768 (3)	0.18667 (15)	0.0392	
O10	0.9132 (2)	0.47855 (18)	0.16574 (10)	0.0420	
C11	0.5358 (2)	0.8483 (2)	0.33392 (12)	0.0251	
C12	0.5418 (2)	0.9877 (2)	0.36769 (12)	0.0266	
C13	0.6550 (3)	1.0751 (2)	0.34846 (13)	0.0315	
C14	0.6565 (3)	1.2033 (2)	0.37762 (14)	0.0370	
C15	0.5459 (3)	1.2459 (2)	0.42665 (15)	0.0406	
C16	0.4328 (3)	1.1595 (2)	0.44577 (15)	0.0402	
C17	0.4305 (3)	1.0309 (2)	0.41629 (13)	0.0344	
C18	0.4604 (2)	0.74727 (19)	0.38447 (11)	0.0253	
C19	0.5194 (3)	0.7164 (2)	0.45543 (12)	0.0322	
C20	0.4554 (3)	0.6179 (3)	0.50012 (13)	0.0405	
C21	0.3303 (3)	0.5504 (2)	0.47385 (16)	0.0422	
C22	0.2698 (3)	0.5832 (2)	0.40451 (16)	0.0397	
C23	0.3342 (3)	0.6812 (2)	0.35977 (13)	0.0312	
H21	0.6217	0.6791	0.2367	0.0282*	
H31	0.6344	0.5365	0.3548	0.0341*	
H81	0.8335	0.6581	0.4126	0.0399*	
H82	0.9062	0.5176	0.3880	0.0400*	
H91	1.0125	0.7635	0.1745	0.0585*	
H92	0.9002	0.8665	0.2083	0.0596*	
H93	0.8509	0.7721	0.1411	0.0587*	
H111	0.4814	0.8523	0.2851	0.0297*	
H131	0.7306	1.0474	0.3158	0.0374*	
H141	0.7337	1.2628	0.3636	0.0445*	
H151	0.5485	1.3315	0.4472	0.0487*	
H161	0.3564	1.1873	0.4788	0.0478*	
H171	0.3527	0.9733	0.4299	0.0420*	
H191	0.6040	0.7642	0.4731	0.0384*	
H201	0.4969	0.5968	0.5471	0.0498*	
H211	0.2866	0.4832	0.5036	0.0514*	
H221	0.1848	0.5371	0.3861	0.0482*	
H231	0.2913	0.7017	0.3118	0.0394*	
H1	0.980 (4)	0.748 (3)	0.3251 (17)	0.0433*	

### Atomic displacement parameters (Å^2^)

**Table:**

	*U*^11^	*U*^22^	*U*^33^	*U*^12^	*U*^13^	*U*^23^
O1	0.0237 (7)	0.0228 (7)	0.0350 (7)	0.0012 (6)	0.0009 (6)	−0.0054 (6)
C2	0.0247 (9)	0.0202 (9)	0.0278 (9)	−0.0005 (8)	0.0007 (8)	−0.0031 (8)
C3	0.0329 (11)	0.0225 (9)	0.0303 (10)	0.0008 (8)	0.0044 (9)	−0.0034 (8)
O4	0.0330 (8)	0.0242 (7)	0.0359 (8)	0.0010 (6)	0.0024 (7)	−0.0046 (7)
C5	0.0280 (10)	0.0300 (10)	0.0324 (10)	0.0045 (9)	−0.0011 (9)	−0.0022 (9)
C6	0.0243 (10)	0.0261 (9)	0.0340 (10)	0.0013 (8)	0.0015 (8)	−0.0012 (9)
N7	0.0264 (9)	0.0306 (9)	0.0404 (10)	0.0011 (8)	−0.0073 (8)	−0.0026 (8)
C8	0.0375 (12)	0.0306 (11)	0.0331 (11)	0.0063 (10)	−0.0040 (10)	0.0005 (9)
C9	0.0358 (12)	0.0361 (12)	0.0457 (13)	−0.0005 (10)	0.0122 (11)	0.0078 (11)
O10	0.0462 (10)	0.0399 (9)	0.0398 (9)	0.0087 (8)	0.0086 (8)	−0.0084 (8)
C11	0.0224 (9)	0.0270 (9)	0.0260 (9)	0.0031 (8)	−0.0017 (8)	−0.0001 (8)
C12	0.0285 (10)	0.0247 (9)	0.0265 (9)	0.0045 (8)	−0.0011 (8)	0.0008 (8)
C13	0.0307 (11)	0.0267 (10)	0.0372 (11)	0.0034 (9)	0.0025 (10)	0.0025 (9)
C14	0.0385 (12)	0.0246 (10)	0.0478 (13)	−0.0016 (10)	0.0034 (11)	0.0045 (10)
C15	0.0496 (15)	0.0239 (11)	0.0482 (14)	0.0065 (10)	0.0007 (12)	−0.0049 (9)
C16	0.0421 (14)	0.0335 (12)	0.0452 (13)	0.0062 (11)	0.0100 (11)	−0.0051 (10)
C17	0.0361 (12)	0.0289 (11)	0.0383 (12)	0.0023 (10)	0.0079 (10)	−0.0004 (9)
C18	0.0265 (10)	0.0230 (9)	0.0265 (10)	0.0029 (8)	0.0018 (9)	−0.0030 (8)
C19	0.0408 (13)	0.0287 (10)	0.0272 (10)	0.0053 (11)	−0.0017 (10)	−0.0039 (8)
C20	0.0570 (16)	0.0362 (12)	0.0283 (10)	0.0138 (11)	0.0069 (12)	0.0020 (10)
C21	0.0474 (14)	0.0292 (11)	0.0501 (14)	0.0055 (11)	0.0209 (13)	0.0051 (10)
C22	0.0348 (12)	0.0299 (11)	0.0544 (15)	−0.0019 (10)	0.0093 (12)	−0.0035 (11)
C23	0.0284 (10)	0.0295 (10)	0.0356 (10)	0.0009 (8)	0.0004 (9)	−0.0038 (9)

### Geometric parameters (Å, °)

**Table:**

O1—C2	1.414 (2)	C12—C13	1.390 (3)
O1—C11	1.439 (2)	C12—C17	1.389 (3)
C2—C3	1.538 (3)	C13—C14	1.387 (3)
C2—C6	1.537 (3)	C13—H131	0.935
C2—H21	0.992	C14—C15	1.388 (4)
C3—O4	1.471 (2)	C14—H141	0.951
C3—C8	1.519 (3)	C15—C16	1.382 (4)
C3—H31	0.968	C15—H151	0.934
O4—C5	1.358 (3)	C16—C17	1.393 (3)
C5—C6	1.524 (3)	C16—H161	0.945
C5—O10	1.200 (3)	C17—H171	0.941
C6—N7	1.495 (3)	C18—C19	1.393 (3)
C6—C9	1.497 (3)	C18—C23	1.389 (3)
N7—C8	1.486 (3)	C19—C20	1.390 (4)
N7—H1	0.89 (3)	C19—H191	0.954
C8—H81	0.986	C20—C21	1.397 (4)
C8—H82	0.971	C20—H201	0.932
C9—H91	0.955	C21—C22	1.377 (4)
C9—H92	0.983	C21—H211	0.942
C9—H93	0.968	C22—C23	1.389 (4)
C11—C12	1.523 (3)	C22—H221	0.954
C11—C18	1.512 (3)	C23—H231	0.952
C11—H111	0.990		
			
C2—O1—C11	114.34 (15)	C12—C11—C18	113.84 (17)
O1—C2—C3	114.94 (16)	O1—C11—H111	107.6
O1—C2—C6	109.83 (16)	C12—C11—H111	108.5
C3—C2—C6	91.88 (16)	C18—C11—H111	108.3
O1—C2—H21	113.2	C11—C12—C13	120.81 (19)
C3—C2—H21	112.4	C11—C12—C17	120.2 (2)
C6—C2—H21	112.8	C13—C12—C17	119.0 (2)
C2—C3—O4	100.99 (16)	C12—C13—C14	120.3 (2)
C2—C3—C8	101.98 (17)	C12—C13—H131	119.9
O4—C3—C8	106.51 (17)	C14—C13—H131	119.8
C2—C3—H31	116.9	C13—C14—C15	120.6 (2)
O4—C3—H31	113.0	C13—C14—H141	119.7
C8—C3—H31	115.7	C15—C14—H141	119.7
C3—O4—C5	106.11 (16)	C14—C15—C16	119.4 (2)
O4—C5—C6	106.84 (17)	C14—C15—H151	120.5
O4—C5—O10	122.5 (2)	C16—C15—H151	120.2
C6—C5—O10	130.6 (2)	C15—C16—C17	120.2 (2)
C2—C6—C5	99.24 (16)	C15—C16—H161	120.3
C2—C6—N7	104.02 (17)	C17—C16—H161	119.5
C5—C6—N7	100.82 (17)	C16—C17—C12	120.6 (2)
C2—C6—C9	119.55 (18)	C16—C17—H171	119.2
C5—C6—C9	116.07 (19)	C12—C17—H171	120.3
N7—C6—C9	114.42 (19)	C11—C18—C19	120.3 (2)
C6—N7—C8	104.77 (16)	C11—C18—C23	120.47 (19)
C6—N7—H1	106 (2)	C19—C18—C23	119.2 (2)
C8—N7—H1	115 (2)	C18—C19—C20	120.4 (2)
C3—C8—N7	102.82 (18)	C18—C19—H191	119.1
C3—C8—H81	110.3	C20—C19—H191	120.5
N7—C8—H81	111.3	C19—C20—C21	119.7 (2)
C3—C8—H82	111.0	C19—C20—H201	119.8
N7—C8—H82	109.7	C21—C20—H201	120.5
H81—C8—H82	111.3	C20—C21—C22	119.9 (2)
C6—C9—H91	111.7	C20—C21—H211	120.3
C6—C9—H92	108.6	C22—C21—H211	119.9
H91—C9—H92	107.9	C21—C22—C23	120.4 (3)
C6—C9—H93	110.2	C21—C22—H221	120.3
H91—C9—H93	110.1	C23—C22—H221	119.3
H92—C9—H93	108.2	C18—C23—C22	120.4 (2)
O1—C11—C12	106.90 (17)	C18—C23—H231	120.5
O1—C11—C18	111.45 (16)	C22—C23—H231	119.1

### Hydrogen-bond geometry (Å, °)

**Table:**

*D*—H···*A*	*D*—H	H···*A*	*D*···*A*	*D*—H···*A*
C20—H201···O10^i^	0.93	2.36	3.293 (3)	174
N7—H1···O10^ii^	0.89 (2)	2.52 (3)	3.395 (3)	168

Symmetry codes: (i) −*x*+3/2, −*y*+1, *z*+1/2; (ii) −*x*+2, *y*+1/2, −*z*+1/2.
